# Faceting of Si and Ge crystals grown on deeply patterned Si substrates in the kinetic regime: phase-field modelling and experiments

**DOI:** 10.1038/s41598-021-98285-1

**Published:** 2021-09-22

**Authors:** Marco Albani, Roberto Bergamaschini, Andrea Barzaghi, Marco Salvalaglio, Joao Valente, Douglas J. Paul, Axel Voigt, Giovanni Isella, Francesco Montalenti

**Affiliations:** 1 L-NESS and Department of Materials Science, University of Milano - Bicocca, 20125 Milan, Italy; 2 L-NESS and Dipartimento di Fisica, Politecnico di Milano, 22100 Como, Italy; 3 Institute of Scientific Computing and Dresden Center for Computational Materials Science, TU Dresden, 01062 Dresden, Germany; 4 James Watt School of Engineering, University of Glasgow, Glasgow, G12 8LT UK

**Keywords:** Computational methods, Semiconductors

## Abstract

The development of three-dimensional architectures in semiconductor technology is paving the way to new device concepts for various applications, from quantum computing to single photon avalanche detectors. In most cases, such structures are achievable only under far-from-equilibrium growth conditions. Controlling the shape and morphology of the growing structures, to meet the strict requirements for an application, is far more complex than in close-to-equilibrium cases. The development of predictive simulation tools can be essential to guide the experiments. A versatile phase-field model for kinetic crystal growth is presented and applied to the prototypical case of Ge/Si vertical microcrystals grown on deeply patterned Si substrates. These structures, under development for innovative optoelectronic applications, are characterized by a complex three-dimensional set of facets essentially driven by facet competition. First, the parameters describing the kinetics on the surface of Si and Ge are fitted on a small set of experimental results. To this goal, Si vertical microcrystals have been grown, while for Ge the fitting parameters have been obtained from data from the literature. Once calibrated, the predictive capabilities of the model are demonstrated and exploited for investigating new pattern geometries and crystal morphologies, offering a guideline for the design of new 3D heterostructures. The reported methodology is intended to be a general approach for investigating faceted growth under far-from-equilibrium conditions.

## Introduction

In the last decades, materials research in different fields, such as microelectronics and photonics, has heavily focused on miniaturization. In more recent years, both the scientific and industrial interest has progressively included in mainstream materials research the development of three-dimensional (3D) heterostructures allowing for functionalization, diversification and optimization rather than the reduction of scale^1–11^. Patterning emerged as a powerful and straightforward technique to control the position of the 3D structures. Also, it proved effective in determining the shape of micro- and nanostructures grown on patterned substrates^12–15^, or patterned at some intermediate stage during growth. Understanding and controlling the interplay between the top-down patterning and the bottom-up self-assembly processes, returning a given crystal morphology, is complex^16^, especially due to the far-from-equilibrium growth conditions typically required to achieve the 3D growth. The well-established concept of equilibrium crystal shape^17^, determined by the Wulff construction^18,19^ and depending only on the surface-energy density, is not applicable. Indeed, the kinetics of adatom redistribution on the crystal surface plays a major role in determining the orientation of the crystal facets that develop during growth. A possible approach to describe the crystal shape evolution under kinetic growth conditions is the so-called Borgstrom construction^20^, which traces the motion of a faceted profile by assigning a fixed growth rate for each crystal facet and translating each facet accordingly. This treatment is, however, over-simplified as it does not provide any description of intra- and inter-facet diffusion resulting from non-uniform material supply and competition between neighbouring facets for incorporation.

The influence of adatom kinetics is particularly relevant for the specific case of the epitaxial growth of vertical homo- or heterostructures^21^, discussed in this article. In particular, it has been demonstrated that, by exploiting high deposition rates and relatively low growth temperatures, it is possible to integrate Ge microcrystals on Si deeply patterned substrates^22,23^. These microcrystals can either grow vertically aligned, remaining separated by gaps as narrow as a few tens of nanometers, or merge into a suspended layer^24,25^. The major advantages of this technique are the capability of fully relaxing the thermal strain^26,27^ and of expelling the threading dislocations out of the crystal sidewalls^28^. It was also demonstrated^29^ how dislocations can even be completely avoided by growing SiGe graded layers, thus making of the pillar pattern a promising platform for the dislocation-free integration of Ge on Si, alternative to other approaches in the literature^30–32^. Moreover, this growth strategy proved to be successful also in the hetero-integration of GaAs^33–35^, 3C-SiC^36–38^, and GaN^39^ on Si pillars.

The focus of this paper is on the morphological evolution of Si and Ge vertical microcrystals grown on patterned Si substrates. Such microstructures may be relevant for the development of photodetectors in the infrared range, which are of technological interest in autonomous driving, night vision and industrial automation applications. Taking control of the 3D faceting is fundamental for the applications, as it can alter light propagation and absorption^40,41^ within the micro-crystal, where the electron–hole pairs are generated. While a first attempt to treat non uniform growth across the micropillar facets was reported in Refs.^23,42^, such a description remains qualitative as being limited to two-dimensions (2D) and lacks an in-depth treatment of the incorporation and diffusion kinetics.

Recently, a phase-field model for the kinetic growth of crystals has been developed^21^ and it has been successfully applied to the study of homoepitaxial growth of GaAs fins by selective area epitaxy^43^ and of the formation of a suspended 3C-SiC layer on Si pillars^36^. In this paper, the model is adapted for the simulation of the 3D faceting of vertical microstructures made of Si and Ge, grown on deeply patterned Si substrates. This is a significant extension as, so far, only 2D simulations in the high symmetry^42^ {110} cross-sections have been reported to characterize the growth of these structures, with no possibility to tackle the full complexity of the multifaceted 3D shape.

The work is organized as follows. First, the details of the experimental procedure are explained, and the phase-field model is described, highlighting the features that are crucial to simulate this peculiar, highly out-of-equilibrium growth on deeply patterned substrates. Then, the kinetic coefficients controlling the facet-dependent adatom incorporation rates, hardly computable e.g. by ab-initio methods, are estimated by a reverse-engineering procedure on a set of targeted experiments. Finally, the model is applied to investigate and predict some 3D effects during the growth of vertical microstructures, in close comparison with the experimental data.

## Methods

### Experiments

While a wide literature on Ge or SiGe micro-crystals growth on deeply-patterned Si substrates already exists^23,24,27,28,42,44–46^, the homoepitaxial case of Si/Si is far less documented and the existing data are insufficient to yield a quantitative characterization of the growth process. Extended growth experiments have been performed for this latter case. The substrates used in the following experiments were fabricated by patterning standard Si wafers through either optical or electron beam lithography, followed by dry etching in an inductively-coupled plasma reactive ion etch (ICP RIE) tool. Before the deposition, the substrates were cleaned by a Radio Corporation of America (RCA) cleaning process, followed by a dip in a 5% HF solution to remove the native silicon oxide. The depositions were performed by Low-Energy Plasma-Enhanced Chemical Vapor Deposition (LEPECVD). This technique employs a high density–low energy Ar plasma to grow high-quality materials at a lower temperature compared to typical thermal processes, while maintaining high deposition rates^47^. Moreover, such rates are only dependent on the plasma parameters, thus allowing for the independent control of the deposition rate (approximately 5 nm/s for this set of experiments) and substrate temperature during growth (700 °C for Si, 560 °C for Ge).

**Figure 1:**
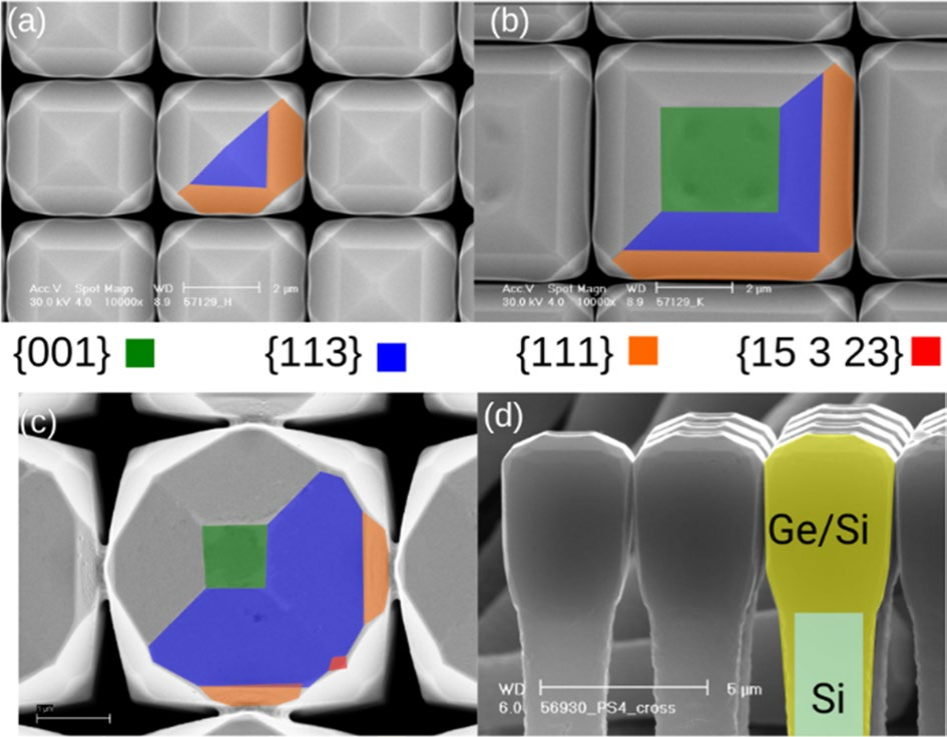
SEM-images of the growth experiments. A top view for Si microcrystals on top of (**a**) 1 × 1 μm Si pillars and (**b**) of 4 × 4 μm Si pillars, both spaced by 3 μm. (**c**) A top view for a Ge microcrystal. Facets are identified by the colors. (**d**) A schematic diagram of the growth on top of the patterned Si pillar.

In Fig. 1 scanning electron microscope (SEM) views of the Si (panels (a) and (b)) and Ge microcrystals (panel (c)) are presented. The top facets of the Si micro-crystals consist of {001}, {113} and {111} facets, as labeled in Fig. 1. Notice that the (001) surface is present only in wider pillars in panel (b), while it is not visible in smaller ones in panel (a), despite the same growth conditions have been used in both cases. The crystal is bounded by {110} vertical sidewalls (visible in panel (d)). The Si crystals tend to fill all the available space in the square pattern, leaving a nanometric gap as already observed for the Ge case^23^. The same set of facets also appears in Ge crystals, as shown in panel (c), with a stronger tendency to expose {113} facets. Additionally, some other facets may appear for Ge crystals at the corners of the square shape, which are addressed here as {15 3 23} planes^42^. Nonetheless, it is worth noting that the actual facet may be a vicinal one, or a set of vicinal facets, which are difficult to distinguish by the experimental techniques available, so that the attribution of this Miller index is to be considered as an average estimate.

The experiments discussed in this paper are performed at 700 °C for Si, and 560 °C for Ge. Such a difference is meant to balance their different diffusion coefficients in order to guarantee a comparable epitaxial growth at high deposition rates for both materials.

### Phase-field kinetic growth model

The morphology of the microcrystals grown by LEPECVD is mainly determined by two factors: material deposition from the gas phase to the crystal surface and surface diffusion. A continuum approach is selected to account for both the size of the system, on the scale of micrometers, and the time scale of the growth process, of the order of tens of minutes. This implies that local, atomic-scale effects are coarse-grained, even if they enter effectively in the parameter estimates. Moreover, as the scope of the model is to reproduce the overall evolution of the faceted profile, the dynamics of nucleation, growth and coalescence of each layer within the facets is neglected^48,49^. The main focus here is the far-from-equilibrium growth regime resulting from the competition between facets, similarly to Refs.^42,50,51^, with the aim of simulating the full kinetic pathway of the morphological evolution of the micro-crystals in 3D. To this purpose, the phase-field^52,53^ approach developed in Ref.^21^ is exploited. It consists of a diffused domain method, tracing the morphology of the crystal implicitly, through an order parameter $\varphi \left({\varvec{x}}\right)$, with ${\varvec{x}}\in\Omega$ and $\Omega$ a simple parallelepipedal domain, including both the solid phase, where $\varphi =1$, and the surrounding vacuum region, where $\varphi =0$. There is a smooth transition region in between that is well described by

$$\varphi \left({\varvec{x}}\right)=\frac{1}{2}\left[1-\text{tanh}\left(\frac{3d\left({\varvec{x}}\right)}{\epsilon }\right)\right]$$

where $\epsilon$ is the thickness of the interface between the two phases and $d\left({\varvec{x}}\right)$ is the signed distance of a point ${\varvec{x}}\in{\varvec{\Omega}}$ from the crystal surface, which is nominally identified by the $\varphi =0.5$ isoline. No distinction is made within the solid region between the growing crystal and the substrate, both in the case of Si/Si homoepitaxy and in the case of Ge/Si heteroepitaxy, assuming that the Si pillars have the only role of setting the initial shape for the crystal growth. Si-Ge mixing effects are also neglected due to the fast-deposition regime. The evolution of the system is determined by the combined effect of deposition and surface diffusion. The former is set by the orientation-dependent growth rate $F\left(\widehat{{\varvec{n}}}\right)$, that will be detailed further in the following Section. The latter is driven by the local gradient of the chemical potential $\mu$. More precisely, the evolution equation for $\varphi$ reads:

1 $$\frac{\partial \varphi }{\partial t}=F\left(\widehat{{\varvec{n}}}\right)\left|\nabla \varphi \right|+\nabla \cdot M\left(\varphi \right)\nabla \mu$$

with $\widehat{{\varvec{n}}}= -\nabla \varphi /|\nabla \varphi |$ being the local profile orientation as given by the outward-pointing normal of the surface of the solid phase, $F(\widehat{{\varvec{n}}})$ the amount of material that impinges on a particular point at the surface, as a function of the local surface orientation $\widehat{{\varvec{n}}}$, and $M\left(\varphi \right)$ the mobility function. Under the assumption that only surface diffusion occurs, $M\left(\varphi \right)=\left(36{M}_{0}/\epsilon \right){\varphi }^{2}{\left(1-\varphi \right)}^{2}$ is set^54,55^, to confine the evolution within the diffused free-surface region, with ${M}_{0}$ the diffusivity constant, taken as an average for all facets for simplicity. Without additional kinetic effects, the chemical potential is defined as the functional derivative of the system free energy $\mathcal{F}$: ${\mu }_{eq}=\delta \mathcal{F}/\delta \varphi$. For the present analysis, the only contribution to the free-energy is the surface energy. This holds true also in the case of Ge/Si heteroepitaxial growth as the misfit strain is fully relaxed by defects since the initial growth stages (as compared to the micrometric growth considered here). Then, in the phase-field formulation^52^, the approximation of the surface energy reads

$$\mathcal{F}\left[\varphi \right]={\int }_{\Omega }\left[ \gamma \left(\frac{\epsilon }{2}{\left|\nabla \varphi \right|}^{2}+\frac{1}{\epsilon }B\left(\varphi \right)\right)\right]d{\varvec{x}}$$

with $B\left(\varphi \right)=18{\varphi }^{2}{\left(1-\varphi \right)}^{2}$ a double-well potential, and $\gamma$ the surface energy density, in general dependent on the surface orientation $\widehat{{\varvec{n}}}$, but here treated as a constant as detailed below (see also Ref.^43,56^). Then the equilibrium chemical potential can be defined as:

$${\mu }_{eq}=-\epsilon \gamma {\nabla }^{2}\varphi +\frac{1}{\epsilon }\gamma { B}^{^{\prime}}\left(\varphi \right)$$

Due to the high deposition rates used in experiments, it is required to tackle far-of-equilibrium growth conditions^23^. Therefore, following the seminal work by Cahn and Taylor^57^ and its numerical treatment^58^, an additional kinetic term is to be considered in the definition of the chemical potential $\mu$, accounting for the dynamics of material rearrangement on the surface, which is controlled by an effective facet-dependent incorporation time $\tau \left(\widehat{{\varvec{n}}}\right)$^21^:

2 $$\mu ={\mu }_{eq}+\epsilon \tau \left(\widehat{{\varvec{n}}}\right)\frac{\partial \varphi }{\partial t}$$

Following Ref.^59^, the orientation dependent incorporation time is conveniently defined as:

$$\tau \left(\widehat{{\varvec{n}}}\right)={\sum }_{i}{\tau }_{i}{\left(\widehat{{\varvec{n}}}\cdot {\widehat{{\varvec{m}}}}_{i}\right)}^{{w}_{i}}\Theta \left(\widehat{{\varvec{n}}}\cdot {\widehat{{\varvec{m}}}}_{i}\right)$$

with $i$ indexing each set of {hkl} facets, ${\widehat{{\varvec{m}}}}_{i}$ the normal direction of the observed crystal facets (i.e. the orientations along which the incorporation time has local maxima ${\tau }_{i}$) and the exponent ${w}_{i}$ a parameter controlling the width of each maximum. At variance with the thermodynamic properties (e.g. the surface energy density), these kinetic parameters are difficult to be inferred a-priori, e.g. by ab-initio methods, as inherently involving the out-of-equilibrium character of the growth process. Thus, they are determined by an alternative empirical strategy exploiting a reverse-engineering approach, based on the comparison with experimental data, as successfully demonstrated in Refs.^43,60–62^. More precisely, the relative ratios between the incorporation coefficients are estimated by matching the simulation profiles with those observed for a set of experimental test cases. This procedure is detailed in the Model calibration section. Following the approach proposed in Ref.^43^, the only orientation dependent property considered here is the incorporation time $\tau \left(\widehat{{\varvec{n}}}\right)$, while the surface energy $\gamma$ is assumed to be isotropic, meaning that the faceting is fully driven by $\tau \left(\widehat{{\varvec{n}}}\right)$. This assumption is reasonable due to the strong out-of-equilibrium character of the growth, where the kinetics of material rearrangement at the surface tends to dominate. This is in agreement with the previous literature, as all the 2D simulations proposed so far did not model the anisotropic surface energy, but considered assigned growth velocities of the facets and a kinetic growth regime^21,42^. On the contrary, the surface energy anisotropy would be required to simulate the annealing dynamics^63^. The assumption of kinetic faceting is crucial for the development of the numerical code as it significantly reduces the computational cost of the 3D simulations.

The profile evolution dictated by the two second-order partial differential equations (1) and (2) is solved numerically by finite element method via the toolbox AMDiS^64,65^. A semi-implicit time integration scheme is used. For numerical reasons^66^, a stabilizing function $g\left(\varphi \right)\sim B\left(\varphi \right)$ is introduced as a multiplicative factor of $\mu$ in the left-hand side of Eq. (2). The micrometer has been chosen as the unit length of the simulation domain, so to match the size of the epitaxial crystals under investigation. In particular, $\epsilon =0.2$ μm is used, with a local refinement that guarantees a mesh spatial resolution of ~ 30 nm in the interface region, sufficient to accurately trace the evolution of the growth front (namely corresponding to 6–7 discretization points) while a coarser resolution is used elsewhere. Neumann boundary conditions for the $\varphi$ function are set on the boundaries of the simulation cell $\Omega$. Parallel execution has been exploited with an iterative solver based on the biconjugate gradient stabilized method^67^. The numerical parameters for the surface energy densities, ${\gamma }^{Si}=9 eV/n{m}^{2}$ and ${\gamma }^{Ge}=6 eV/n{m}^{2}$, are set in agreement with literature values^68^. The coefficients ${\tau }_{i}$ for the incorporation times are determined by the fitting procedure reported in Table 1.

**Table 1:** Incorporation parameters $\uptau \left(\widehat{\mathbf{n}}\right)$ fitted for Si and Ge, normalized with respect to the incorporation time on the {113} facet, with ${\tau }_{\left\{113\right\}}^{\text{Si}}=0.42 \text{eV s}/{\text{nm}}^{4}$ and ${\tau }_{\left\{113\right\}}^{\text{Ge}}=0.28 \text{eV s}/{\text{nm}}^{4}$.

${{\varvec{m}}}_{i}$	Si ${\tau }_{i}$	Ge ${\tau }_{i}$
$\{113\}$	1	1
$\{100\}$	0.43	0.71
$\{111\}$	1.36	0.57
{15 3 23}	–	0.57
$\{110\}$	0.29	0.5

## Model calibration

In order to reliably model the evolution of the crystal morphologies, the parameters controlling the deposition flux distribution $F\left(\widehat{{\varvec{n}}}\right)$ and the incorporation of material at the surface $\tau \left(\widehat{{\varvec{n}}}\right)$ have to be defined. This is performed by exploiting a small set of experimental results for fitting the simulation parameters, to be validated subsequently by the comparison with other experiments. The first experimental data required by the model is the width of the microcrystal, which can be easily measured by images of the top view, such as in Fig. 1, and the height, measurable by a lateral view and deducible from the growth time. From these data, the parameters for the flux operator $F\left(\widehat{{\varvec{n}}}\right)$ of Eq. (1) can be properly set. The parameters are tuned by firstly choosing the magnitude of the ratio between the material deposition rate and the mobility ${F}_{0}/{M}_{0}$. Experimentally, the vertical growth regime can be achieved by a high deposition flux (${F}_{0}=5 nm/s$), such to enforce far-from-equilibrium conditions. In the simulations these are obtained by considering the experimental value for ${F}_{0}$ and setting ${M}_{0}=2.7\cdot {10}^{-5} n{m}^{-6} eV s$ for Si and ${M}_{0}=1.8\cdot {10}^{-5} n{m}^{-6} eV s$ for Ge. Once the magnitude for the ${F}_{0}/{M}_{0}$ ratio is determined, the spatial distribution of the deposition flux has to be specified. The gas distribution in the LEPECVD reactor has been reported^42^ as the combination of a vertical ${f}_{vert}$ and a hemispherical component ${f}_{hs}$: $F\left(\widehat{{\varvec{n}}}\right)={F}_{0}\left({f}_{hs}\left(0.5+0.5\left[\text{0,0},-1\right]\cdot \widehat{{\varvec{n}}}\right)+{f}_{vert}\left[\text{0,0},-1\right]\cdot \widehat{{\varvec{n}}}\right)$, with ${f}_{hs}=1-{f}_{vert}$. The ${f}_{hs}$ contribution alone would be more suitable for the simulation of isolated pillars, as it considers the deposition on the top and on the sides of the micro-crystal as nearly equivalent. When reducing the weight of ${f}_{hs}$, in favour of ${f}_{vert}$, the role of flux shadowing is incremented thus reducing the lateral impingement of material, a condition which is required for the self-limited lateral growth of the micro-crystals.

**Figure 2:**
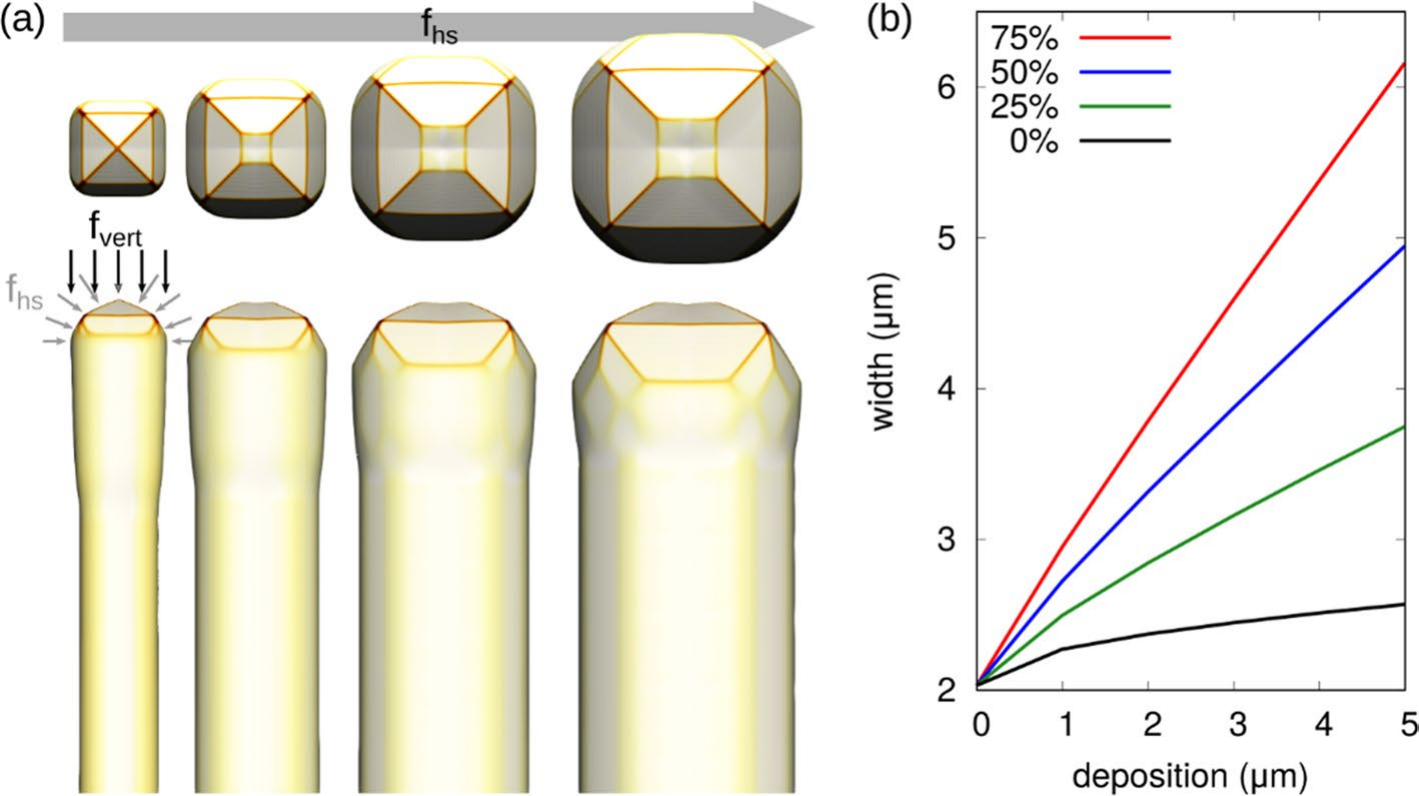
The effect of the distribution of the deposition flux on the crystal facets. (**a**) Simulated morphologies: from left to right, the hemispheric component ${f}_{hs}$ of the flux is increased from 0% up to 75% by 25% increments. 5 μm of Si are deposited along the [00–1] direction. (**b**) A plot of the microcrystal width as a function of the material deposited vertically for different ${f}_{hs}$.

To illustrate and clarify the general effect of the flux distribution, Fig. 2 reports a set of simulation profiles for a given choice of the incorporation times and varying the contribution of ${f}_{vert}$ and ${f}_{hs}$. As expected, by changing the flux toward more isotropic conditions, we obtain an increased lateral size. It can also be noticed, however, how the areas of the {111} and (001) facets are influenced by the change in the flux, although the same set of incorporation coefficients has been used in all simulations. This highlights the correlation existing between the definition of $\tau \left(\widehat{{\varvec{n}}}\right)$ and $F\left(\widehat{{\varvec{n}}}\right)$. With this respect, we notice that simulations including a more detailed description of flux distributions, e.g. shadowing effects^23,69^, may change the material supply within the same facets, thus slightly altering the material redistribution dynamics predicted by the present set of parameters. The implementation of flux shadowing in the phase-field approach^70^ goes beyond the scope of this work as it would require computationally demanding calculations in 3D. In the analysis reported in the following Section, a value of ${f}_{hs}=0.25$ has been used to better reproduce the growth on pillars separated by a 3 μm gap. Notice that, in the experimental settings, ${f}_{vert}$ is expected to change depending on the crystal morphology and thus on the growth stage. In particular, lateral growth is expected to be more pronounced in the initial stages, when the shadowing is not particularly effective due to the initial gap between Si pillars. On the contrary, at later stages, lateral growth is strongly reduced. Therefore, the present approach can be considered as an average behaviour between these two regimes. To extend these simulations to a longer growth time, provided that no merging of adjacent pillars occurs, the lateral deposition should be progressively reduced through control of the ${f}_{hs}$ parameter. Notice that the lateral expansion is not exactly equal to the material deposited laterally by the flux operator, as adatoms can redistribute both towards the top and along the vertical sidewalls.

To reproduce the evolution of the crystal morphology, once the optimal flux ratio has been identified, the incorporation time ${\tau }_{i}$ has to be set for each orientation corresponding to a facet. In the Experiments section, Fig. 1, the key facets have been already identified and their relative size has been discussed. The incorporation rate is generally inversely proportional to the area of the facets: as a general rule, those growing at a slower rate are those developing a larger area. Here these parameters are tuned both for Ge and Si, to obtain a faceting at the top of the microcrystal with the same facet positioning and size hierarchy as observed in the experiments. This is done by iteratively adjusting ${\tau }_{i}$ from an initial guess to obtain ratios of the observed facet areas close to the experimental ones (the peak width ${w}_{i}$ is arbitrary set equal to 100 for Si and to 200 for Ge, to avoid an excessive overlapping of the additional {15 3 23} peaks).

**Figure 3:**
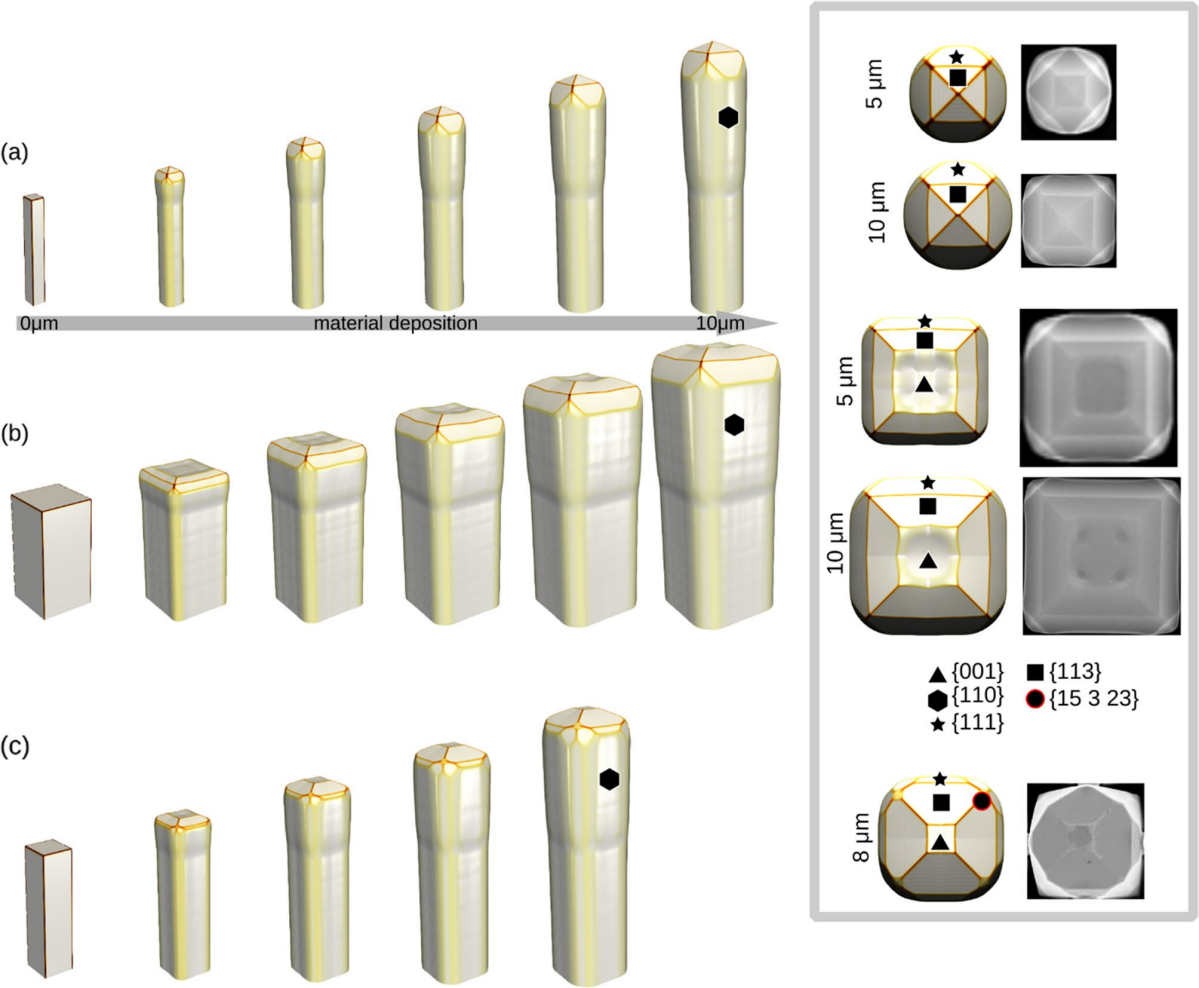
The simulation sequence fitted from the experiments. (**a**) Si deposited on Si pillar 1 × 1 μm, (**b**) Si deposited on Si pillar 4 × 4 μm and (**c**) Ge deposited on Si pillar 2 × 2 μm. On the right the corresponding top view is shown for specific material depositions.

Simulated profiles obtained by the best-fitting parameter set are shown in Fig. 3 for both Si (panels (a) and (b)) and Ge (panel (c)) microcrystals. Here a single crystal is shown, as the interaction with the neighboring microcrystals in the array is effectively included in the flux operator, as discussed above. The advantage of this procedure is that it relies on SEM images, that can be acquired without any complex sample preparation, and on a limited number of experiments. Still, a full growth simulation is required for every guess of the parameter set, as the whole history of the growth is responsible for the final facets, as it will be more extensively discussed in the next Section, when considering template effects. For Si, two sizes of the pillar base (1 μm and 4 μm), and two deposition stages (5 μm and 10 μm thickness of deposited material), have been considered. In particular, the latest stage of 10 μm deposition has been used as the main reference for measuring the facet widths, while the 5 μm growth has been employed to reconstruct the proper evolution of the facets. For instance, the appearance of the (001) facet has been confirmed only on pillars of 4 μm wide base, both at 10 μm and at 5 μm, and its parameters have been chosen accordingly. This observation permits to immediately exclude exceedingly long values of the incorporation time for the (001) orientation, which may still result in an equivalent shape at 10 μm of deposition, but that would make the (001) facet apparent also in microcrystals grown on a 1 μm base pillar. Additionally, it is worth noting that the {110} facets are never observed at the pillar top, neither at 5 μm nor at 10 μm, but only at the crystal sidewalls^23^. This once again restricts the admissible values of incorporation time for the {110} facet to consider in the fitting procedure. For Ge, the data required for the fitting have been extracted directly from the available literature^42^. The optimized values obtained after the fitting procedure are reported in Table 1.

The {15 3 23} facet family has been fitted only for Ge, as it is not observed in Si microcrystals. It must be pointed out that all parameters, in particular mobility and incorporation times, are set on the scale of facets and hence they have to be considered as effective values, averaging any local effect related to the surface roughness recognizable in the experimental views. Notice that in each simulation, the whole set of facets for each {hkl} family is included, not only those that are expected to appear, in order to minimize the bias of the simulation parameters on the outcome. Noticeably, our simulations clearly reproduce the change in the ratio between {113} and {111} facets, when changing from Si to Ge, in good agreement with the experimental observations.

**Figure 4:**
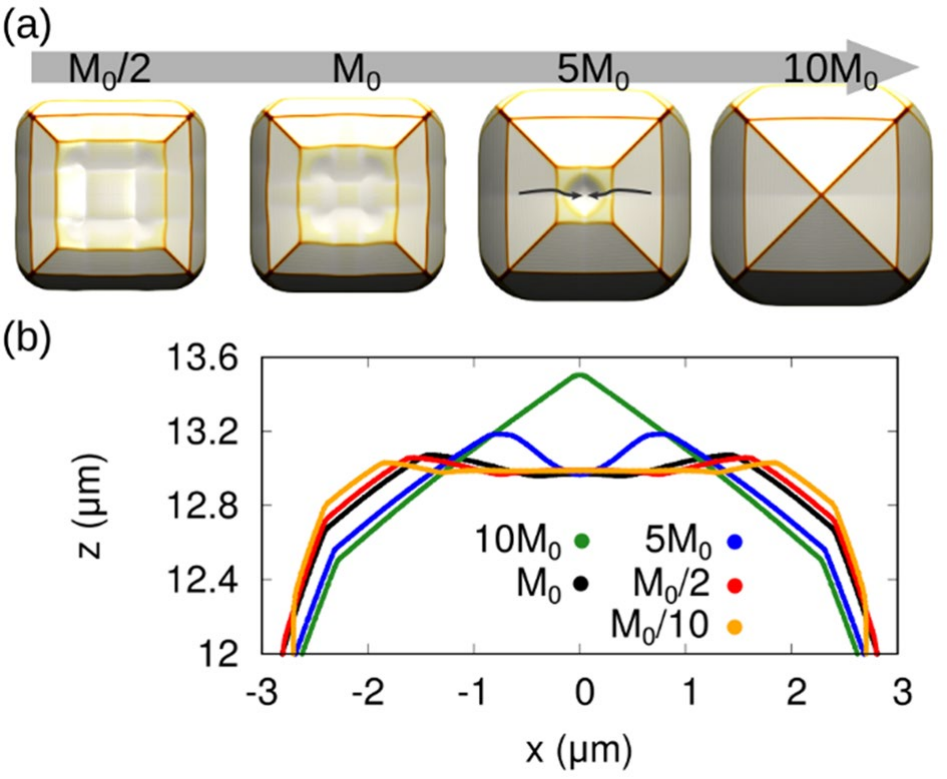
Simulations of the effect of the surface mobility on the faceting. (**a**) Top view of Si microcrystal (5 μm tall). The initial Si width is 4 μm for the growth of Si. (**b**) The cross-sectional surface profile of the simulations in (**a**) highlighting the tendency to transfer material from the {113} to cover the (001) facet.

The amount of material transferred among facets, e.g. from {113} to (001), is controlled mainly by the difference in the incorporation coefficients ${\tau }_{i}$. The mobility coefficient, however, also plays a role in this dynamics. Figure 4a demonstrates the effect of different mobility parameters, keeping the ${F}_{0}/{M}_{0}$ ratio sufficiently high to guarantee the vertical growth. The value of the surface mobility can be finely tuned to guarantee the existence of a limited diffusion length (${F}_{0}$ is chosen as a fixed experimental data). This can change the width of the top (001) facet, as observed also in the experiments^42^, and may eventually return an accumulation of material at the rim of the (001) facet in case of reduced mobility. It is worth noting that this feature (highlighted by the surface profiles in Fig. 4b) is not simply a minor refinement of the model, but as demonstrated in Ref.^71^ it can be responsible for the formation of voids in the microcrystal, due to shadowing related phenomena.

It is important to notice that surface mobility is one of the few parameters that can be controlled experimentally, essentially by changing the growth temperature within the appropriate range to guarantee monocrystalline growth. From an applied perspective, low temperatures are preferred when a wide (001) top is required, while high temperatures favour a more “rounded” shape, eventually leading to merging and to the formation of a continuous suspended layer^63^. For all the other cases presented in this paper, the temperature of 700 °C has been chosen for Si, to compare with the dedicated experiments, while the temperature of 560 °C has been chosen for Ge, allowing for the comparison with the case grown on a (111) substrate that will be discussed in the following (see Fig. 7 and related discussions).

## Understanding based on model predictions

**Figure 5:**
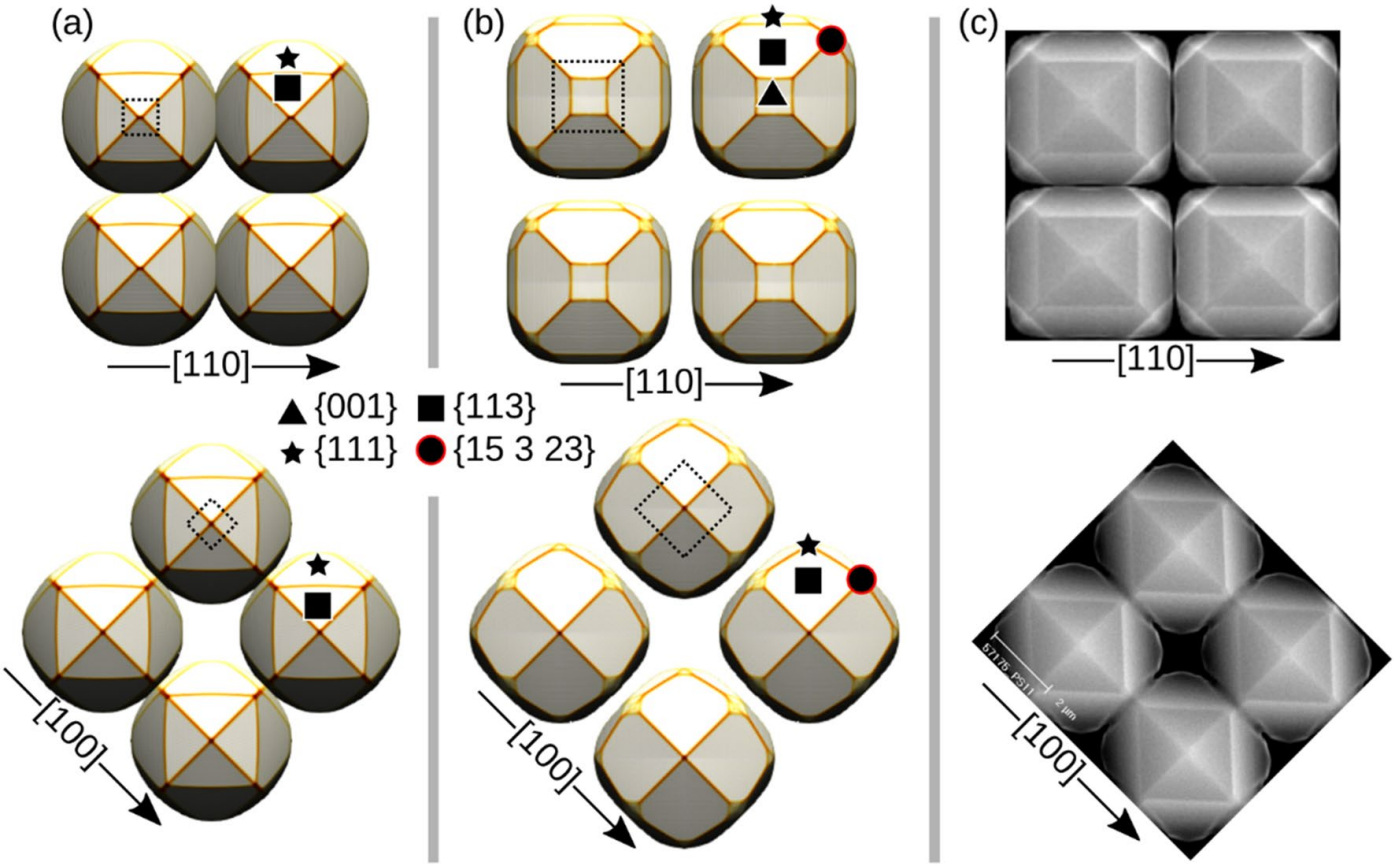
Growth on rotated patterns. The comparison between (**a**) simulations and (**c**) experiments of the growth of 10 μm of Si on two patterns with the same 1 × 1 μm pillar array, but rotated by 45° on the (001) plane so that pillar rows are along [110] and [100] respectively. (**b**) The simulation for Ge on 2 × 2 μm Si pillars. The initial pillar is sketched by the dotted squares, a single pillar is simulated and then mirrored to mimic the array.

After the parameter calibration from the previous Section, the model is fully defined and exploitable to predict specific features of the crystal facet evolution during growth before performing additional experiments. It is important to notice that, in all the following results, no parameter is adjusted, except for setting the different geometries and orientations of the initial Si pillar. The full 3D model considered here is crucial to capture specific features of the vertical growth of the microcrystals. Indeed, while 2D models are capable of fitting the cross-section evolution^42^, reproducing most of the behaviours discussed so far, they miss the proper dynamics of material redistribution as well as any effect due to the actual pillar shape with respect to an infinite ridge. A first case that requires a 3D model is the comparison between microcrystals grown on patterned pillars with [110] or [100] oriented edges. This is easily achieved by rotating by 45° about the [001] axis the lithographic mask used to pattern the Si (001) substrate. The outcome is reported in Fig. 5, where both the SEM experimental images and the corresponding simulation results are shown.

Although the facets appearing at the crystal top are the same for the two cases, the resulting shapes are notably different. In particular, in the [100] case, the initial Si pillar is vertically bounded by {100} facets, instead of {110}. Therefore, the {111} facets that appear during growth cannot lie any more on the pillar sidewalls. Indeed, they start to develop in correspondence of the vertical edges of the initial Si pillar. As a result, they appear to be smaller since the beginning if compared to the case along [110] considered so far. This effect is not limited to the first stages of the growth, but persists during the deposition. Its influence is visible also after 10 μm of deposition, as shown in Fig. 5a. The four {113} facets on the top do not seem to be significantly influenced by the rotation of the pillar, apart from the different boundary with the {111} facets just discussed. In the case of Ge deposition in Fig. 5b, this is even more evident due to the generally faster growth of the {111} facets if compared to the {113}. In the [110]-aligned case the {111} facets are still visible, despite being small, because their formation is initially triggered by the large {110} sidewalls. In contrast, in the [100]-aligned case, {111} facets develop in correspondence of the vertical edges of the Si pillar. Due to their faster incorporation time, they cannot expand, as surface diffusion tends to move material towards them. Therefore, they appear only as small facets at the microcrystal corners. On the contrary, {113} facets expand since the beginning, and they dominate the microcrystal morphology at later deposition stages, even closing the (001) facet. The simulation predictions for Si are confirmed by the experiments shown in Fig. 5c. Indeed, the same effect on the facets, induced by the rotation of the pillars, can be observed from the top-view images. Notice that, in addition to what was discussed so far, the rotation of the pattern also corresponds to a different flux shielding, not considered in the present paper. Still, for the cases here reported, shadowing effects are expected to play a minor role as indicated by the substantial agreement between the present model predictions and the experimentally observed faceting.

**Figure 6:**
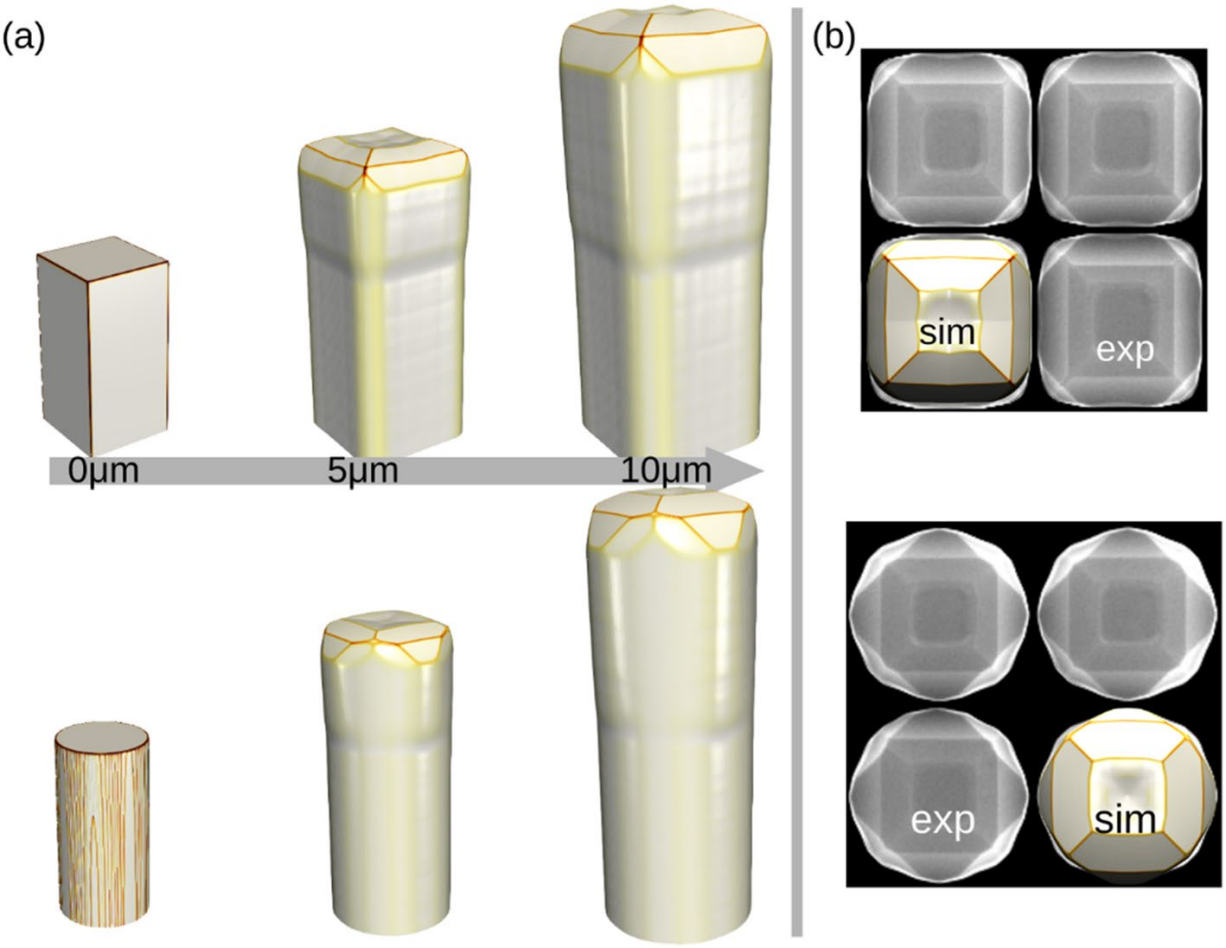
Pillar template effect. (**a**) The evolution of the microcrystals at different deposition stages on Si pillars with different shapes as predicted by the simulations (top: parallelepiped shape; bottom: cylindrical shape) and (**b**) experimentally observed. Top views of the simulated profiles are superposed to the SEM images for comparison. The initial Si pillar is 4 μm wide, and 10 μm of Si is deposited.

The role of the Si pillar geometry in templating the shape of the growing microcrystal can be made more evident by another targeted case. In particular, in Fig. 6a, the growth on a circular pillar is compared with the reference case of a square one. This comparison reveals a clear template effect. In the former condition the facets tend to rearrange in a more rounded shape, as confirmed also by the experimental images in Fig. 6b. The larger is the lateral size of the pillars, or the lower is the diffusion length, the more evident this phenomenon is. The template effect proves, once again, the far-from-equilibrium character of these types of growth, for which the kinetic phase-field model is well suited. The faceted morphologies do not correspond to the equilibrium shape, usually defined by the surface energy densities, but represent a metastable state of the kinetic pathway in which the facets evolve due to material deposition and pattern geometry.

**Figure 7:**
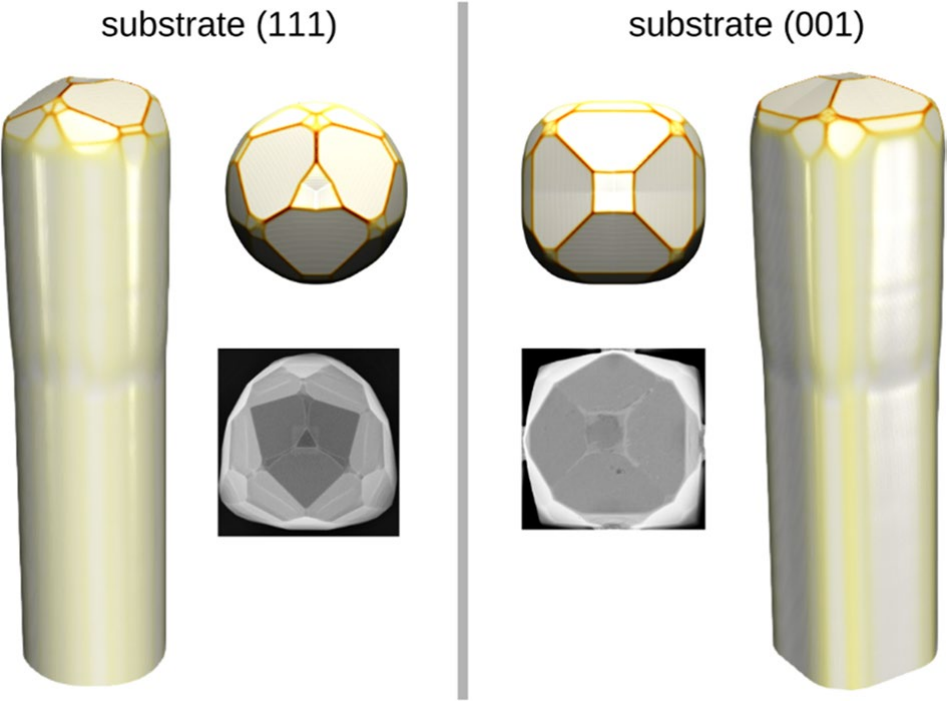
Growth on a (111) substrate. A comparison of the growth of Ge microcrystal (8 μm tall) on a 2 μm wide Si pillar on the (111) (left, circular Si pillar) and (001) (right, square Si pillar) substrate.

Another important degree of freedom that can be exploited to change the emerging crystal facets^72^ is represented by the orientation of the substrate used for the patterning. Indeed, while most of the growths for Ge and Si were performed on (001) substrates, other surfaces, such as the (111), can be exploited to achieve different crystal morphologies. Indeed, this was proven experimentally in a previous work for Ge^22^. Simulation profiles for the Ge growth on (001) and (111) substrates are shown in Fig. 7. The important point is that for both cases the same set of incorporation times is used, although it was fitted only on samples grown on (001) substrates. The only change is in the orientation of the simulation cell. Nevertheless, the results obtained are in good agreement with the experimental data. The simulations predict a different set of facets for the (111) case if compared to the growth on (001). First of all, this is because of the three-fold symmetry of the (111) substrate, compared to the four-fold symmetry of the (001) surface. As a consequence, the facet at the top, which is now a (111) plane, is triangular. The good correspondence between the morphology predicted by the present model and reported in the experiments represents a proof of the reliability of the model in reproducing the growth of semiconductor microcrystals.

**Figure 8:**
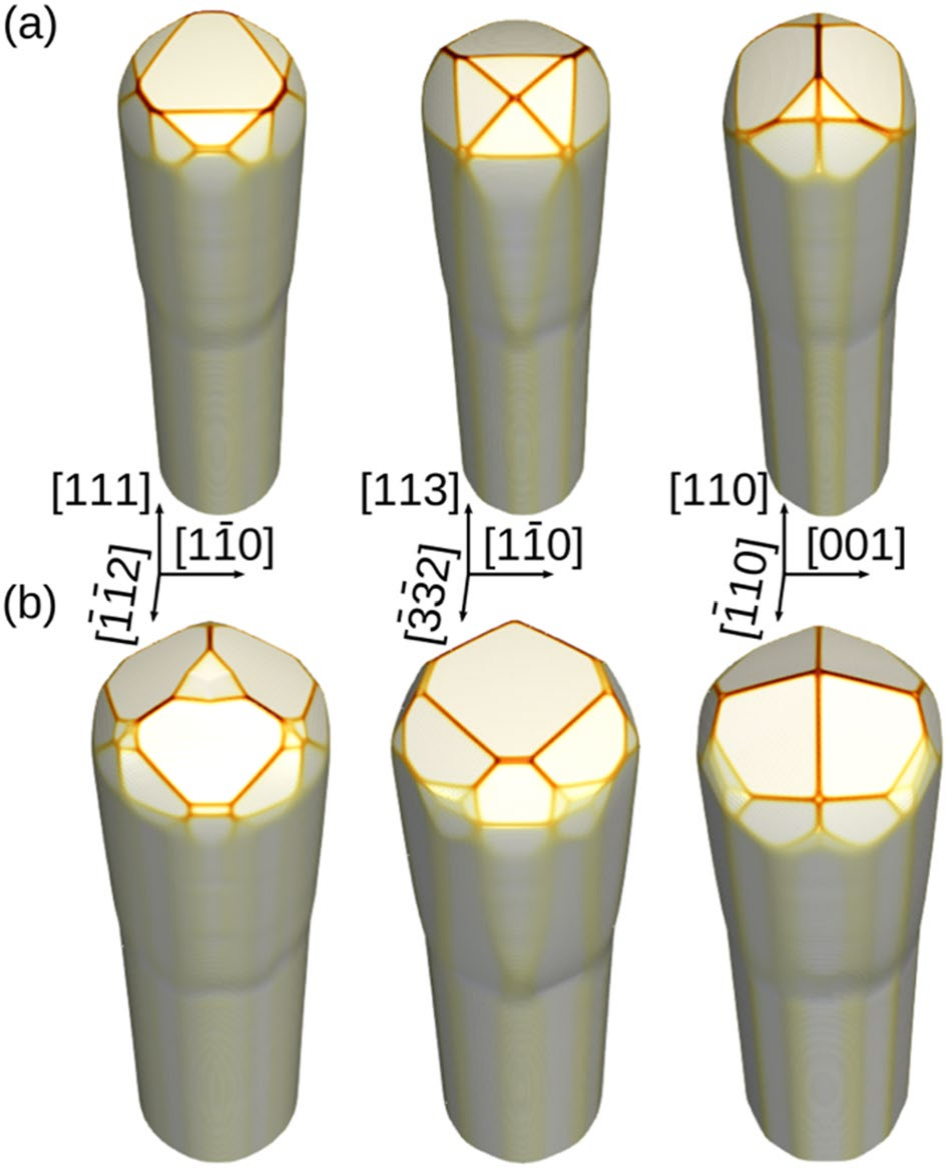
Predictive results for other substrate orientations. A comparison of the facets of (**a**) Si and (**b**) Ge microcrystals grown on circular Si pillars, (**a**) 1 μm wide and (**b**) 2 μm wide, after the deposition of 8 μm, for three different substrate orientations: (111), (113) and (110).

The advantage of having a calibrated simulation tool is that it can be straightforwardly extended to cases beyond the present experiments, for example to predict the possible crystal shapes when using other substrate orientations, such as (113) or (110), both for Si and Ge. The results of this analysis are reported in Fig. 8, where it is possible to highlight both the differences induced on the facets, as well as the different behaviours of Si and Ge. When growing on Si as shown in panel (a), the shape on the (111) substrate is dominated by a flat (111) plane, characterized by the slowest incorporation rate. The (113) substrate result appears very similar by tilting the shape observed on the (001) plane. The (110) case is more asymmetric, with two large {111} facets on one side, and four smaller {113} facets on the other. For Ge, shown in panel (b), due to the different ratio between the incorporation times of {111} and {113} facets, a different behaviour is observed. In particular, for the (111) substrate the shape is dominated by {113} facets, while the {111} facets remain small. As a consequence of the slower growth rate of {113} facets, the shape observed on the (113) plane cannot be described as a simple rotation of the others, as it was for Si, and it is again dominated by the flat {113} top surface. Finally, the (110) substrate, differently from Si, becomes more symmetric, with four large {113} facets and small {110} facets at the top, bounded by even smaller {15 3 23} facets.

## Conclusions

In this paper, the morphology of vertical homo- and heterostructures, grown under kinetic conditions, has been characterized by exploiting a 3D simulation code based on a convenient phase-field model able to predict the actual facet shapes during growth. As explicitly demonstrated, few experiments are sufficient to estimate the full set of incorporation parameters that, despite being effective, are not limited to a specific experiment, as it would be for the assigned growth velocities used in the simplest growth models, but have a more general applicability. Indeed, once the model was calibrated, it correctly reproduces different experimental configurations without requiring any new fitting. In particular, we showed how by a unique set of parameters it is possible to capture several qualitatively different experimental results: different sizes, different facets, different substrate orientations, different temperatures. Then, the versatility of this approach enables to explore new configurations, providing a reliable predictive tool for the 3D facet morphology. This has been shown for orientations of the substrate unexplored in the literature. A further improvement would consist in the explicit implementation of the shadowing contribution in the material deposition dynamics, for studying the conditions leading to vertical-alignment versus merging during growth. Most interestingly, the modelling technique applied here to Si and Ge can be straightforwardly extended to other materials grown in the kinetic regime on various patterns, both at the micro- and nanoscale, provided that a relatively small set of experiments is available to determine the kinetic parameters by reverse-engineering.

## Data Availability

The simulation datasets generated during the current study are available from the corresponding author on reasonable request.
